# Pyroptosis in inflammation-related respiratory disease

**DOI:** 10.1007/s13105-022-00909-1

**Published:** 2022-07-11

**Authors:** Yuanyu Feng, Min Li, Xiaoting Yangzhong, Xifeng Zhang, Anju Zu, Yunjiao Hou, Lin Li, Shibo Sun

**Affiliations:** 1 Department of Pulmonary and Critical Care Medicine, First Affiliated Hospital, Kunming Medical University, No.295, Xichang Road, Wuhua District, Kunming, China; 2 Clinical Medicine, Innovation Class, 2019 Grade, Kunming Medical University, Kunming, China; 3 Pediatrics, One Class, 2020 Grade, Kunming Medical University, Kunming, China

**Keywords:** Pyroptosis, Inflammation, Respiratory diseases, Inflammasomes, Gasdermin, Caspase-1

## Abstract

Pyroptosis is commonly induced by the gasdermin (GSDM) family and is accompanied by the release of inflammatory cytokines such as IL-1β and IL-18. Recently, increasing evidence suggests that pyroptosis plays a role in respiratory diseases. This review aimed to summarize the roles and mechanisms of pyroptosis in inflammation-related respiratory diseases. There are several pathways involved in pyroptosis, such as the canonical inflammasome-induced pathway, non-canonical inflammasome-induced pathway, caspase-1/3/6/7/GSDMB pathway, caspase-8/GSDMC pathway, caspase-8/GSDMD pathway, and caspase-3/GSEME pathway. Pyroptosis may be involved in asthma, chronic obstructive pulmonary disease (COPD), lung cancer, acute lung injury (ALI), silicosis, pulmonary hypertension (PH), and tuberculosis (TB), in which the NLRP3 inflammasome-induced pathway is mostly highlighted. Pyroptosis contributes to the deterioration of asthma, COPD, ALI, silicosis, and PH. In addition, pyroptosis has dual effects on lung cancer and TB. Additionally, whether pyroptosis participates in cystic fibrosis (CF) and sarcoidosis or not is largely unknown, though the activation of NLRP3 inflammasome is found in CF and sarcoidosis. In conclusion, pyroptosis may play a role in inflammation-related respiratory diseases, providing new therapeutic targets.

## Introduction

Pyroptosis is a novel programmed cell death (PCD) form induced by the gasdermin (GSDM) family, accompanied by the secretion of inflammatory cytokines such as IL-1β and IL-18 [18, 63]. When activated, pyroptosis results in the elimination of bacterial, fungal, and viral pathogens through the robust inflammatory response which is indispensable for organismal homeostasis [29]. However, pyroptosis may also be detrimental to the body, now that the deterioration of diseases occurs with pyroptosis. It is reported that pyroptosis plays an essential role in several diseases, such as cancers, atherosclerosis, and so on [57, 65, 79]. Respiratory diseases are considered to account for the increased mortality rate [64, 67]. It is well established that respiratory diseases are closely related to inflammation [30, 48]. Increasing evidence indicates that pyroptosis is crucial for inflammatory diseases [65]. Recently, accumulative studies focus on the role of pyroptosis in the inflammatory diseases of the lung [89, 91]. This review aimed to summarize the roles and plausible mechanisms of pyroptosis in inflammatory diseases of the lung.

## Molecular mechanism of pyroptosis

As an original PCD form, pyroptosis was discovered after apoptosis and necrosis [79]. In 1992, Zychlinsky et al. first observed this new death form, which was mistakenly depicted as apoptosis in *Shigella flexneri*-infected macrophages [94]. Later, this new cell death form was termed pyroptosis by Brennan following the observation of *Salmonella*-mediated death in macrophages [14]. The characteristics of pyroptosis, to a certain extent, are analogous to those of apoptosis, such as nuclear pyknosis and DNA fragmentation. Meanwhile, pyroptosis is also accompanied by some necrosis-like features, including cellular swelling, pore formation, and membrane disruption [57]. A large body of evidence suggests that the GSDM family leads to the formation of membrane channels and then pyroptosis [63]. The GSDM family consists of six members in humans (GSDMA, GSDMB, GSDMC, GSDMD, GSDME which is also called DFNA5, and GSDMF which is also known as DFNB59 or PJVK) [73]. It is reported that GSDMA, GSDMB, GSDMC, GSDMD, and GSDME are closely related to pyroptosis [73]. Most members of the GSDM family share the highly conserved N-terminal domain (NTD) and C-terminal domain (CTD) [93]. The NTD is activated by the dissociation of CTD to form an inner pore on the membrane which interacts with the cytosol and extracellular space directly [38], allowing the leakage of intercellular contents and the inflow of extracellular water. Accordingly, pyroptosis is redefined as the GSDM-mediated PCD [63].

Increasing studies suggest that various signaling pathways participate in pyroptosis [63, 79].

### Inflammasome-induced signaling pathway

**Fig. 1:**
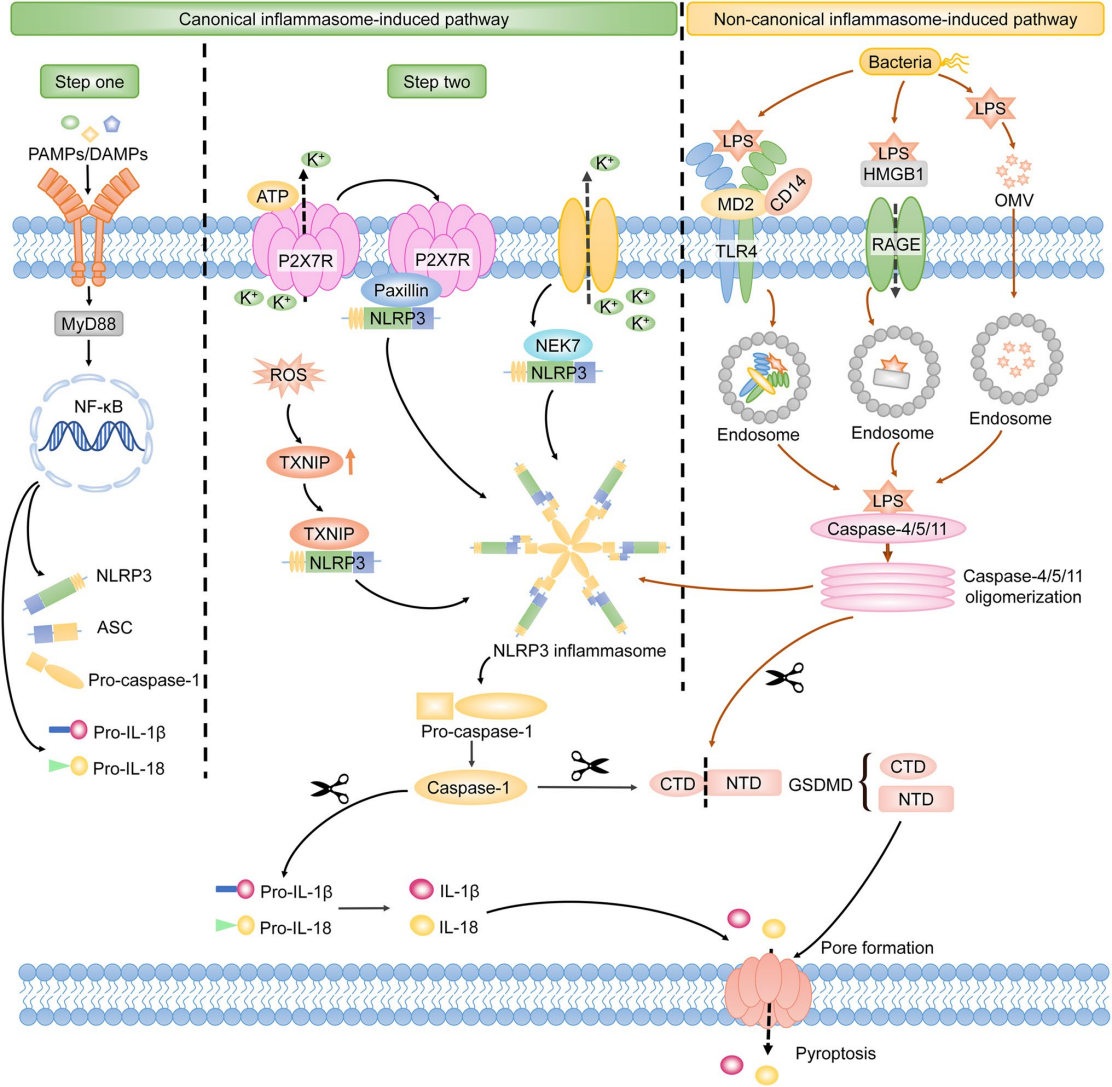
Canonical and non-canonical inflammasome-induced pathways in pyroptosis. The NLRP3 inflammasome is mostly highlighted in the canonical inflammasome-induced pathway. The NLRP3 inflammasome is activated depending on two steps. Step 1 is that the PAMPs or DAMPs stimulate the TLRs/MyD88/NF-κB signaling pathway, leading to the upregulated transcription of NLRP3, ASC, pro-caspase-1, pro-IL-1β, and pro-IL-18. In step 2, the formation of the P2X7R-paxillin-NLRP3 complex activates the NLRP3 inflammasome, which depends on the ATP/P2X7R axis-induced potassium efflux. Simultaneously, NEK7 interacts with NLRP3 in response to the declined intercellular potassium, which contributes to the activation of the NLRP3 inflammasome. In addition, increased TXNIP can also activate the NLRP3 inflammasome by directly binding to NLRP3. The activated NLRP3 inflammasome excites caspase-1. Sequentially, GSDMD is cleaved by caspase-1 and releases GSDMD-NTD to form the membrane pore. Then pyroptosis occurs. Meanwhile, IL-1β and IL-18 are activated by caspase-1 and secreted from the GSDMD-induced pore, which results in the inflammatory cascade downstream. LPS is transferred into the cytosol by the formation of an endosome, which is induced by TLR4/MD2/CD14 complex, HMGB1/RAGE signaling pathway, or OMVs. Intracellular LPS directly activates caspase-4/5/11. Activated caspase-4/5/11 cleaves GSDMD to induce pore formation and pyroptosis. During this procedure, caspase-4/5/11 activates the NLRP3 inflammasome by inducing potassium efflux. Note: MyD88, myeloid differentiation factor 88; NF-κB, nuclear factor kappa-B; ASC, apoptosis-associated speck-like protein containing a caspase-activation and recruitment domain; P2X7R, P2X7 receptor; NEK7, NIMA-related kinase 7; TXNIP, thioredoxin interacting protein; GSDMD, gasdermin D; NTD, N-terminal domain; HMGB1, high mobility group box 1; RAGE, receptor for advanced glycation end-products; OMVs, outer membrane vesicles

Pyroptosis is originally mediated by the inflammatory caspases, which are activated by the inflammasomes [29]. Inflammasomes are multi-protein complexes that contain certain pattern-recognition receptors (PRRs) [29]. Pyroptosis-related PRRs include membrane-bound Toll-like receptors (TLRs), the nucleotide-binding domain and leucine-rich repeat-containing receptors (NLRs), and the absent in melanoma (AIM)-like receptors (ALRs) [33, 79]. These PRRs sense pathogen-associated molecular patterns (PAMPs) and danger-associated molecular patterns (DAMPs) when host cells suffer from abnormal conditions such as microbial infection, stress, and tissue damage, which lead to the activation of inflammasomes [33]. Subsequently, activated inflammasomes lead to the cleavage of inflammatory caspases, which allows the activation of GSDMD [38]. Consequently, the GSDMD-NTD oligomerizes to form the membrane channel, and then pyroptosis occurs [38]. In light of the different involvement of inflammasomes and distinct kinds of inflammatory caspases, the pyroptotic signaling pathways are divided into canonical and non-canonical inflammasome-induced pathways [43] (Fig. 1).

#### Canonical inflammasome-induced pathway

Canonical inflammasomes include AIM2, NLRP1, NLRP3, NLRC4, and pyrin inflammasomes [33, 56]. Canonical inflammasomes play an important role in the canonical inflammasome-induced pathway. They are composed of the sensor acting as PRRs, the effector acting as the pro-caspase-1, and the adaptor acting as the apoptosis-associated speck-like protein containing a caspase-activation and recruitment domain (ASC) which connects the sensor and the effector [56]. The canonical inflammasomes are activated by distinct PAMPs or DAMPs. The AIM2 inflammasome is only activated by cytoplasmic double-stranded DNA (dsDNA) [33]. In addition, NLRP1 and NLRC4 inflammasomes are specially activated by the anthrax lethal toxin and bacterial flagellin, respectively [3, 46]. Meanwhile, the assembly of NRLP3 is triggered by diverse PAMPs, including bacterial pathogens and viral DNA, as well as DAMPs involving ATP, reactive oxygen species (ROS), uric acid crystals, and silica [80]. Currently, more researchers are focusing on the mechanisms of NLRP3 inflammasome in pyroptosis. The NLRP3 inflammasome is activated depending on two steps [80, 84]. Step 1 is that the PAMPs or DAMPs stimulate the TLRs/MyD88/NF-κB signaling pathway, leading to the upregulated transcription of NLRP3, ASC, and pro-caspase-1 [80]. Then, step 2 is the assembly and activation of the NLRP3 inflammasome. In step 2, paxillin recruits NLRP3 to form the P2X7 receptor (P2X7R)-paxillin-NLRP3 complex, which is dependent on the potassium efflux induced by the ATP/P2X7R signaling pathway, leading to the activation of NLRP3 inflammasome [76]. Simultaneously, the NIMA-related kinase 7 (NEK7) facilitates the oligomerization of ASC by interacting with NLRP3 in response to the intercellular decline of potassium concentration [23]. Recently, it has been reported that the thioredoxin-interacting protein (TXNIP) mediated by ROS plays an important role in the activation of the NLRP3 inflammasome. Sufficient ROS may activate endoplasmic reticulum stress (ERS). When ERS is initiated, phosphorylation of protein kinase R-like endoplasmic reticulum kinase (PERK), which is an ER transmembrane sensor, leads to the activation of eukaryotic translation-initiation factor 2α (eIF2α). Activated eIF2α upregulates the expression of CCAAT-enhancer-binding protein homologous protein (CHOP). The activation of the PERK/eIF2α/CHOP signaling pathway results in the upregulation of TXNIP, which activates the NLRP3 inflammasome by directly interacting with NLRP3 [80]. Accordingly, paxillin, NEK7, and TXNIP are important molecules in the activation of the NLRP3 inflammasome. The activated NLRP3 inflammasome excites caspase-1 depending on the ASC speck. Similar to the NLRP3 inflammasome, the AIM2 and pyrin inflammasomes activating caspase-1 are dependent on ASC speck. NLRP1 and NLRC4 inflammasomes activate caspase-1 independent of the ASC speck [3, 46]. Active caspase-1 cleaves GSDMD to promote the formation of the membrane pore [38]. Meanwhile, caspase-1 cleaves pro-IL-1β and pro-IL-18 into mature IL-1β and IL-18 [18]. Subsequently, IL-1β and IL-18 are secreted from the GSDMD-induced pore, resulting in the inflammatory cascade downstream. It is confirmed that IL-1β is involved in various inflammatory diseases via binding to IL-1 receptor and IL-18 participates in the regulation of both T helper cell 1 (Th1)-type and Th2-type inflammatory cytokines [17, 72] (Fig. 1).

#### Non-canonical inflammasome-induced pathway

Human caspase-4/5 and murine caspase-11 are referred to as non-canonical inflammasomes [28, 43]. It is reported that intracellular lipopolysaccharide (LPS) activating caspase-4/5/11 plays a critical role in the non-canonical inflammasome-induced pathway of pyroptosis [79, 84]. The extracellular LPS is transferred into cells via TLR4/MD2/CD14 receptor-mediated endocytosis [45] or with the help of high mobility group box 1 protein and the receptor for advanced glycation end-products (RAGE) [43, 84], or through outer membrane vesicles (OMVs) [84]. The intracellular LPS will then directly interact with caspase-4/5/11. The activation of caspase-4/5/11 results in the cleavage of GSDMD independent of caspase-1 [28]. Thus, pyroptosis occurs. Different from the canonical inflammasome-induced pathways which is caspase-1-dependent, activated caspase-4/5/11 does not process the precursors of IL-1β and IL-18 but still leads to the secretion of these two cytokines via the GSDMD-NTD channel [28]. Additionally, it is reported that the LPS-activated caspase-11 triggers the activation of the NLRP3 inflammasome by inducing potassium efflux [84]. Accordingly, there may be crosstalk between the canonical and non-canonical inflammasome-induced pathways of pyroptosis (Fig. 1).

### Other signaling pathways

**Fig. 2:**
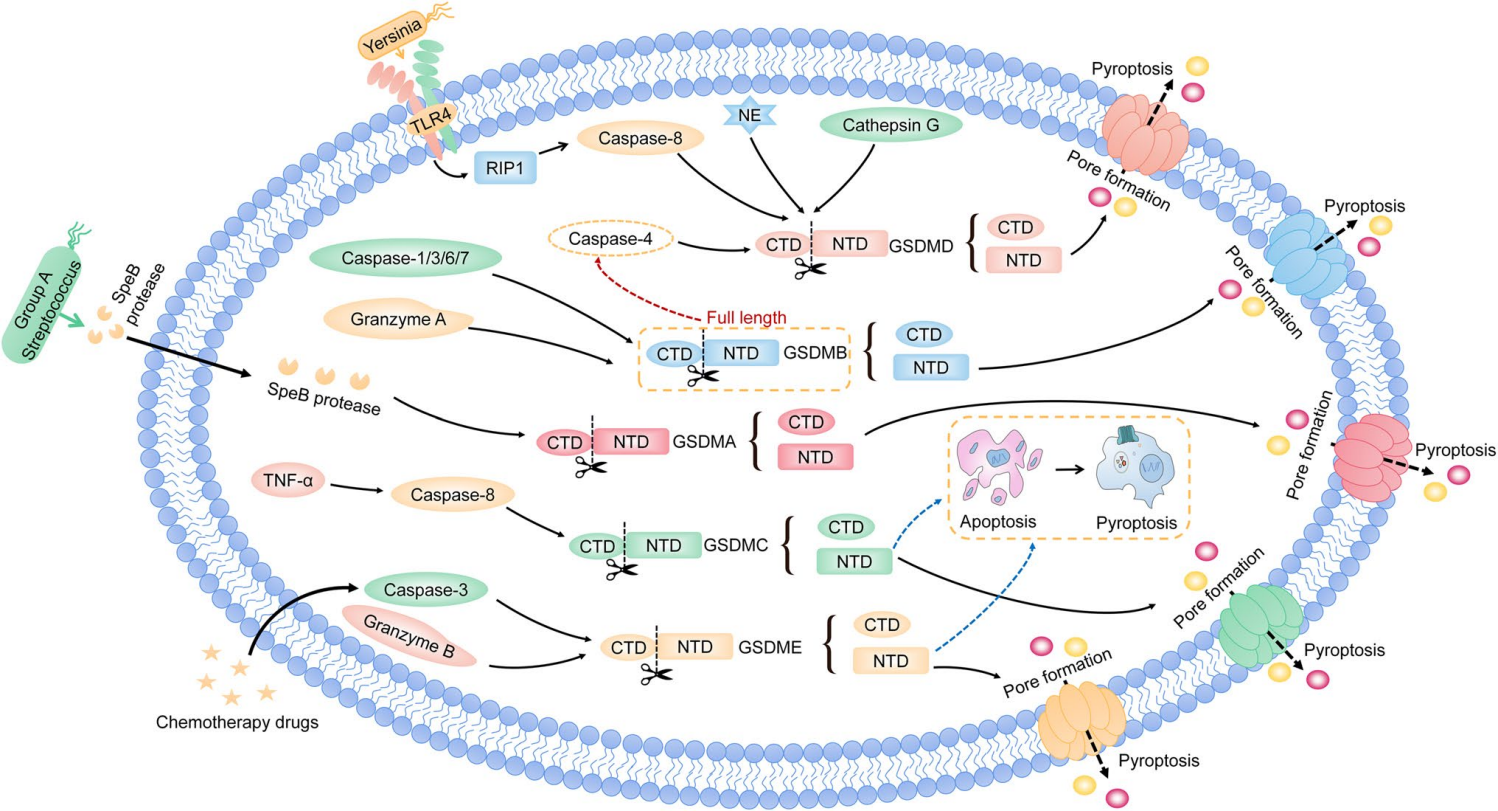
Other signaling pathways in pyroptosis. Most members of the GSDM family play an important role in the pyroptotic signaling pathways. GSDMD can be cleaved by NE, cathepsin G, and caspase-8, which is activated by *Yersinia* via the TLR4/RIP1 signaling pathway to induce pyroptosis. GSDMB is cleaved by caspase-1/3/6/7 and granzyme A to trigger pyroptosis. Meanwhile, the full length of GSDMB enhances caspase-4 cleaving GSDMD to undergo pyroptosis. The cleavage of GSDMC is executed by caspase-8 to induce pyroptosis. GSDME is cleaved to induce pyroptosis with chemotherapy drugs that initiate caspase-3. Additionally, GSDME can be processed by granzyme B to initiate pyroptosis. Moreover, the caspase-8/GSDMC and caspase-3/GSDME signaling pathways can switch apoptosis to pyroptosis. Note: GSDMD, gasdermin D; NE, neutrophil elastase; RIP1, receptor interacting protein 1; GSDMB, gasdermin B; GSDMA, gasdermin A; GSDMC, gasdermin C; GSDME, gasdermin E

Apart from the inflammasome-induced pathway, there are other signaling pathways in pyroptosis. In the other signaling pathways, GSDM family-induced pyroptosis is independent of the inflammasomes and the activation of the GSDM family plays a critical role in pyroptosis [63]. Currently, the members of the GSDM family, including GSDMA, GSDMB, GSDMC, GSDMD, and GSDME, lead to pyroptosis [93] (Fig. 2).

GSDMD is the first member of the GSDM family found to execute pyroptosis [28]. Recently, it is reported that caspase-8, which is activated by *Yersinia* via TLR4/receptor-interacting protein 1 signaling pathway, exhibits cleaving GSDMD to drive pyroptosis [60]. Furthermore, neutrophil elastase and cathepsin G can also activate GSDMD, which results in pyroptosis [73].

In addition, GSDMA is widely expressed in the upper gastrointestinal tract [93]. Though its domains are similar to GSDMD, the active protease of GSDMA is largely unknown. Recently, it is reported that the GSDMA is specifically cleaved by the SpeB protease of *group A Streptococcus* to trigger pyroptosis in keratinocytes [16]. However, whether GSDMA can be activated by other proteases remains unclear.

Different from other GSDM family members, both the cleaved and full-length GSDMB can lead to pyroptosis [93]. It is reported that GSDMB is cleaved by the caspase-1/3/6/7 as well as granzyme A to undergo pyroptosis [73, 92]. While the full-length GSDMB enhances caspase-4 cleaving GSDMD, indirectly promoting non-canonical inflammasome-induced pyroptosis [11].

Meanwhile, the cleavage of GSDMC is executed by caspase-8 to induce pyroptosis. In addition, the upregulation of GSDMC mediated by programmed death-ligand 1 (PD-L1) switches apoptosis to pyroptosis in cancer cells [24].

GSDME is highlighted in cancer for its suppressive activity on the tumor. It is suggested that GSDME is cleaved to induce pyroptosis with chemotherapy drugs that initiate caspase-3 [77]. In addition, the apoptosis induced by caspase-3 is turned on to undergo pyroptosis in the GSDME-positive cells. Accordingly, the caspase-3/GSDME signaling pathway balances the switch between apoptosis and pyroptosis [77]. Moreover, GSDME is cleaved by granzyme B to produce GSDME-NTD, and then pyroptosis is triggered by GSDME [73].

Collectively, most GSDMs play an essential role in pyroptosis. However, further research into the specific mechanism and signaling pathways of this pore-forming protein-induced pyroptosis is warranted in the future.

## Pyroptosis and inflammation-related respiratory diseases

### Pyroptosis and asthma

Asthma is a common chronic respiratory disease that affects more than 300 million individuals worldwide [67]. The main characteristics of asthma include allergic airway inflammation, airway hyperresponsiveness (AHR), and airway remodeling.

Disruption of bronchial epithelial cell (BEC) barrier is important in the pathogenesis of asthma [70, 91]. Damaged epithelia fail to provide mucociliary clearance and allow various allergens to affect the lung [70]. Meanwhile, loss of epithelial integrity leads to the recruitment of inflammatory cells by releasing cytokines and chemokines [70]. It is reported that exposure to environmental allergens such as Dermatophagoides farina 1 or toluene diisocyanate triggers pyroptosis in BECs by activating the NLRP3/caspase-1/GSDMD signaling pathway and induces airway inflammation [70, 91]. Selective inhibition of NLRP3 inflammasome effectively prevents airway allergic inflammation and AHR [30, 91]. Accordingly, NLRP3/caspase-1/GSDMD-induced pyroptosis of BECs may be involved in the deterioration of asthma. Additionally, GSDMB is an executioner of pyroptosis and is related to the pathogenesis of asthma. The increased level of GSDMB is found in asthmatics [15], and the upregulated expression of GSDMB in BECs increases the susceptibility to asthma [39]. These results suggest that GSDMB-induced pyroptosis may play an important role in asthma.

Macrophages are the main effectors against environmental particles. Inflammation mediated by macrophages via pyroptosis may be an important promoter in the development of asthma [59]. It is reported that perfluoroalkyl substances induce GSDMD-dependent pyroptosis in macrophages by activating the AIM2 inflammasome, which aggravates asthmatic inflammation by enhancing the secretion of IL-4 and IL-1β [75]. Prostaglandin E2 (PGE2) plays a protective role in asthma by inhibiting bronchoconstriction and inflammation [86]. It is reported that PGE2 suppresses pyroptosis in macrophages via inhibiting the IFN-β/signal transducer and activator of transcription 1 (STAT1)/caspase-11/GSDMD signaling pathway, which protects the host from allergic airway inflammation [86]. Furthermore, inhibiting pyroptosis of macrophages reduces airway inflammation and airway remodeling in LPS-induced asthmatic rats via suppressing the NLRP3/caspase-1/GSDMD axis [12].

**Fig. 3:**
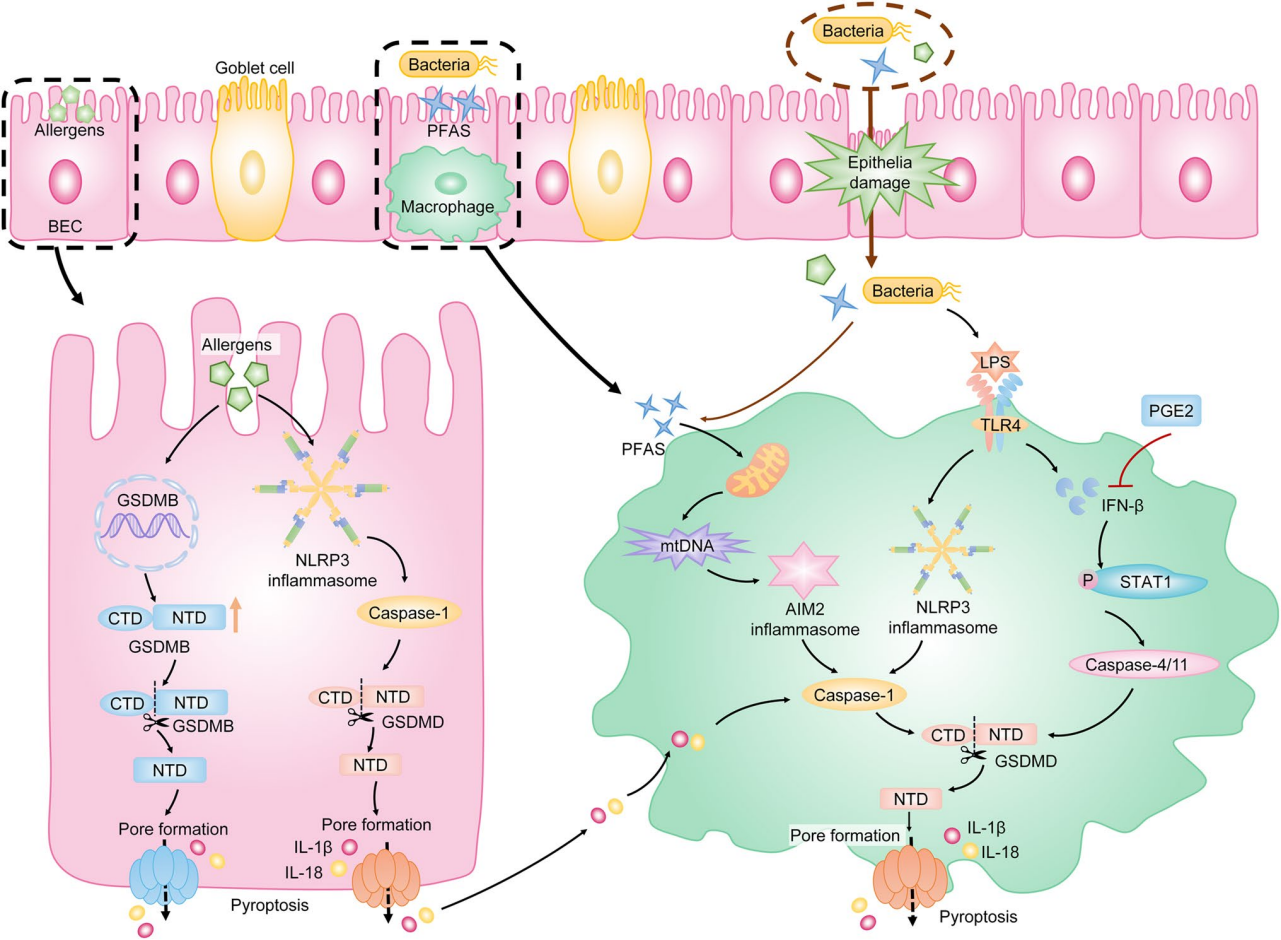
Pyroptosis in asthma. Environmental allergens activate the NLRP3/caspase-1/GSDMD signaling pathway to trigger pyroptosis of BECs. Meanwhile, allergens induce the increased transcription of GSDMB, which results in GSDMB-dependent pyroptosis. Pyroptosis of BECs leads to the loss of epithelial integrity and increased secretion of pro-inflammatory cytokines such as IL-1β and IL-18, which promote augmented and persistent pulmonary inflammation downstream. Additionally, macrophages are the main effectors against environmental particles. PFAS triggers AIM2 inflammasome-induced pyroptosis of macrophages by causing mitochondrial damage, which results in the accumulation of mtDNA. Additionally, bacteria induce pyroptosis of macrophages by activating the TLR4/NLRP3/caspase-1/GSDMD pathway. Meanwhile, LPS causes the upregulation of IFN-β, which results in the activation of STAT1. The activation of STAT1 leads to the increased caspase-4/11, which is important in the pyroptotic signaling pathway. PGE2 indirectly inhibits the caspase-4/11-induced pyroptosis in macrophages by suppressing IFN-β to take a protective role in asthma. Note: BECs, bronchial epithelial cells; PFAS, perfluoroalkyl substance; mtDNA, mitochondrial DNA; AIM2, absent in melanoma 2; IFN-β, interferon-beta; STAT1, signal transducer and activator of transcription 1; PGE2, prostaglandin E2

In summary, these studies directly or indirectly indicate that pyroptosis may play a crucial role in asthma. However, the specific mechanism of pyroptosis in asthma needs to be explored (Fig. 3).

### Pyroptosis and chronic obstructive pulmonary disease

Chronic obstructive pulmonary disease (COPD), regarded as the third most lethal disease in the world, is characterized by consistent airway inflammation and irreversible airflow obstruction [74]. The persistent airway inflammation causes airway remodeling, which is characterized by sloughing of BECs, mucus hypersecretion, and abnormal deposition of collagen [37]. A large body of evidence suggests that cigarette smoke (CS) is the predominant risk factor for COPD [47, 89].

Currently, it is shown that CS extract (CSE) activates the NLRP3/caspase-1/GSDMD signaling pathway and sequentially induces pyroptosis in BECs, which causes the impaired BEC barrier and severe lung injury [74]. Inhibiting the NLRP3 inflammasome results in the downregulation of GSDMD, which causes pyroptosis, as well as the alleviation of pulmonary inflammation [47, 89]. Furthermore, accumulating studies suggest that pyroptosis is activated by CS-induced ERS via ROS and participates in the progression of COPD [41, 89]. CS induces the generation of sufficient ROS to activate ERS and mitochondrial dysregulation [41]. Sequentially, ERS-induced activation of the PERK/eIF2α/CHOP signaling pathway results in increased TXNIP. TXNIP is an important molecule in pyroptosis by activating the NLRP3 inflammasome. It is reported that inhibition of ROS suppresses pyroptosis in CS-treated BEC via inhibiting the ROS/TXNIP/NLRP3/caspase-1 pathway, which leads to the amelioration of COPD both in vitro and vivo [7, 41, 89]. Thus, CS may induce pyroptosis to deteriorate COPD through activating the ROS/TXNIP/NLRP3/caspase-1 signaling pathway.

**Fig. 4:**
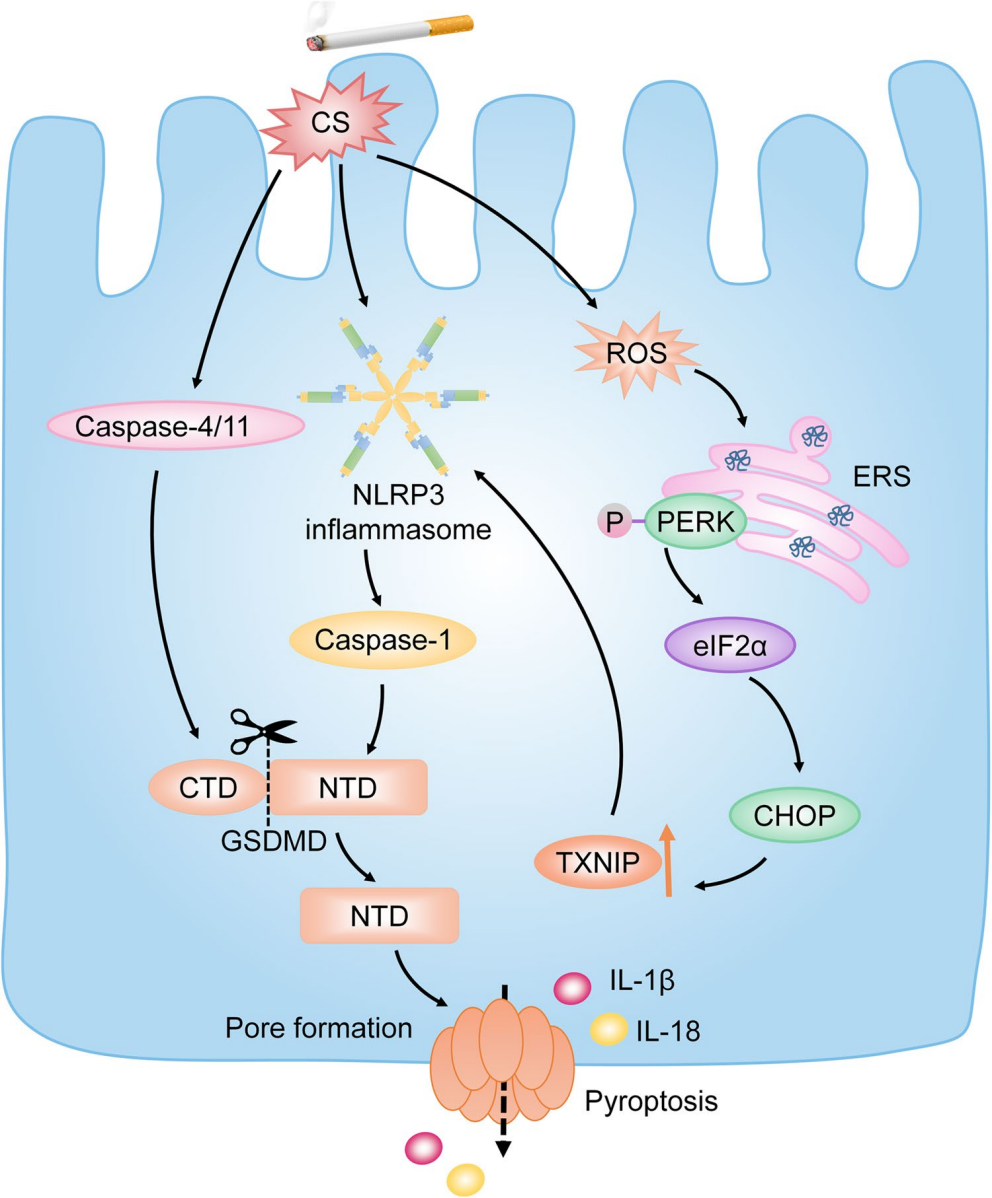
Pyroptosis in COPD. CS is the predominant risk factor for COPD. CS triggers pyroptosis of BECs by activating the NLRP3/caspase-1/GSDMD signaling pathway. Meanwhile, CS induces ERS to trigger pyroptosis of BECs via mediating the generation of sufficient ROS. The initiation of CS-induced ERS results in the activation of PERK, which sequentially upregulates TXNIP by activating the PERK/eIF2α/CHOP pathway. Increased TXNIP contributes to the activation of the NLRP3 inflammasome. Consequently, pyroptosis occurs. Additionally, CS induces caspase-4/11-induced pyroptosis in BECs and accelerates airway remodeling. Note: COPD, chronic obstructive pulmonary disease; CS, cigarette smoke; BECs, bronchial epithelial cells; ERS, endoplasmic reticulum stress; PERK, protein kinase R-like endoplasmic reticulum kinase; TXNIP, thioredoxin interacting protein; eIF2α, eukaryotic translation-initiation factor 2α; CHOP, CCAAT-enhancer-binding protein homologous protein

Additionally, caspase-4, the human analog of murine caspase-11, may be partially responsible for the airway inflammation in COPD [13]. It is reported that caspase-11 knockout in mice treated with CS results in the reduced bronchial deposition of collagen and mucus hypersecretion, which are related to airway remodeling [13]. Therefore, caspase-4/11-induced pyroptosis may participate in CS-induced COPD via the activation of non-canonical inflammasome-induced pathway. However, the specific signaling pathway remains unclear (Fig. 4).

### Pyroptosis and lung cancer

Lung cancer is one of the malignant tumors with the highest morbidity and mortality [64]. Long-term exposure to risk factors results in the continuous activation of the inflammatory response, while chronic inflammation plays an essential role in lung cancer. Inflammasomes play a critical role in the host inflammatory response. It is reported that increased NLRP3 inflammasome, AIM2 inflammasome, caspase-1, IL-1β, and IL-18 are found in lung cancer cells [31]. Notably, the expression of NLRP3 inflammasome is significantly upregulated in high-grade lung adenocarcinoma patients who have a higher risk of progression and postoperative recurrence [31]. NLRP3 inflammasome, AIM2 inflammasome, and caspase-1 are important molecules in pyroptosis. Therefore, pyroptosis may play an important role in lung cancer. It is shown the higher expression of GSDMD, which is the classical executioner of pyroptosis, is associated with larger tumor sizes and more advanced tumor-node-metastasis stage in non-small cell lung cancer (NSCLC) patients. Depletion of GSDMD attenuates the proliferation of NSCLC cells and restricts the growth of tumor cells by facilitating caspase-3-induced apoptosis [21]. These studies suggest that pyroptosis may promote tumor growth by providing an inflammatory microenvironment [79].

However, pyroptosis may also participate in the elimination of cancer tissues by triggering tumor cell death. Many potent anti-tumor drugs can trigger pyroptosis to inhibit the growth of tumors. It is reported that the NLRP3/caspase-1/GSDMD-dependent pyroptosis is enhanced in NSCLC cells treated with polyphyllin VI, leading to the elimination of tumor cells [68]. Meanwhile, cucurbitacin B induces pyroptosis to inhibit the growth of NSCLC cells by activating the TLR4/NLRP3/caspase-1/GSDMD signaling pathway [85]. Moreover, it is demonstrated that p53, which is a tumor suppressor gene, induces pyroptosis to attenuate the proliferation of NSCLC cells by activating the NLRP3/ASC/caspase-1 signaling pathway [90]. Additionally, GSDME, which is regarded as a potential tumor suppressor, is confirmed to execute pyroptosis after the cleavage of caspase-3. It is shown that the NSCLC patients with lower expression of GSDME in tumor tissues are inclined to have a higher mortality rate after platinum treatment [49]. Further research suggests that the GSDME-induced pyroptosis may enhance the sensitivity of cisplatin to NSCLC cells [49]. Meanwhile, it is reported that the increased level of GSDME activated by caspase-3 with chemotherapeutic drugs such as cisplatin and paclitaxel leads to the switch from apoptosis to pyroptosis in lung cancer cells [49, 87]. Accordingly, GSDME-induced pyroptosis may play an important role in lung cancer. Notably, GSDME expression is higher in normal tissues such as the gastrointestinal tract and kidney than in cancer tissues [77, 93]. It is reported that caspase-3/GSDME-dependent pyroptosis mediated by cisplatin occurs in normal tissue and produces chemotherapy side effects during the cisplatin treatment, while inhibition of the caspase-3/GSDME-mediated pyroptosis enhances the tolerance of normal tissue cells to cisplatin [25]. Therefore, it is suggested that caspase-3/GSDME-mediated pyroptosis may be involved in the cisplatin-based side effects such as digestive tract reactions, intestinal injury, and nephrotoxicity [25], which may provide a novel insight into the prevention of cisplatin-based side effects [25, 87].

**Fig. 5:**
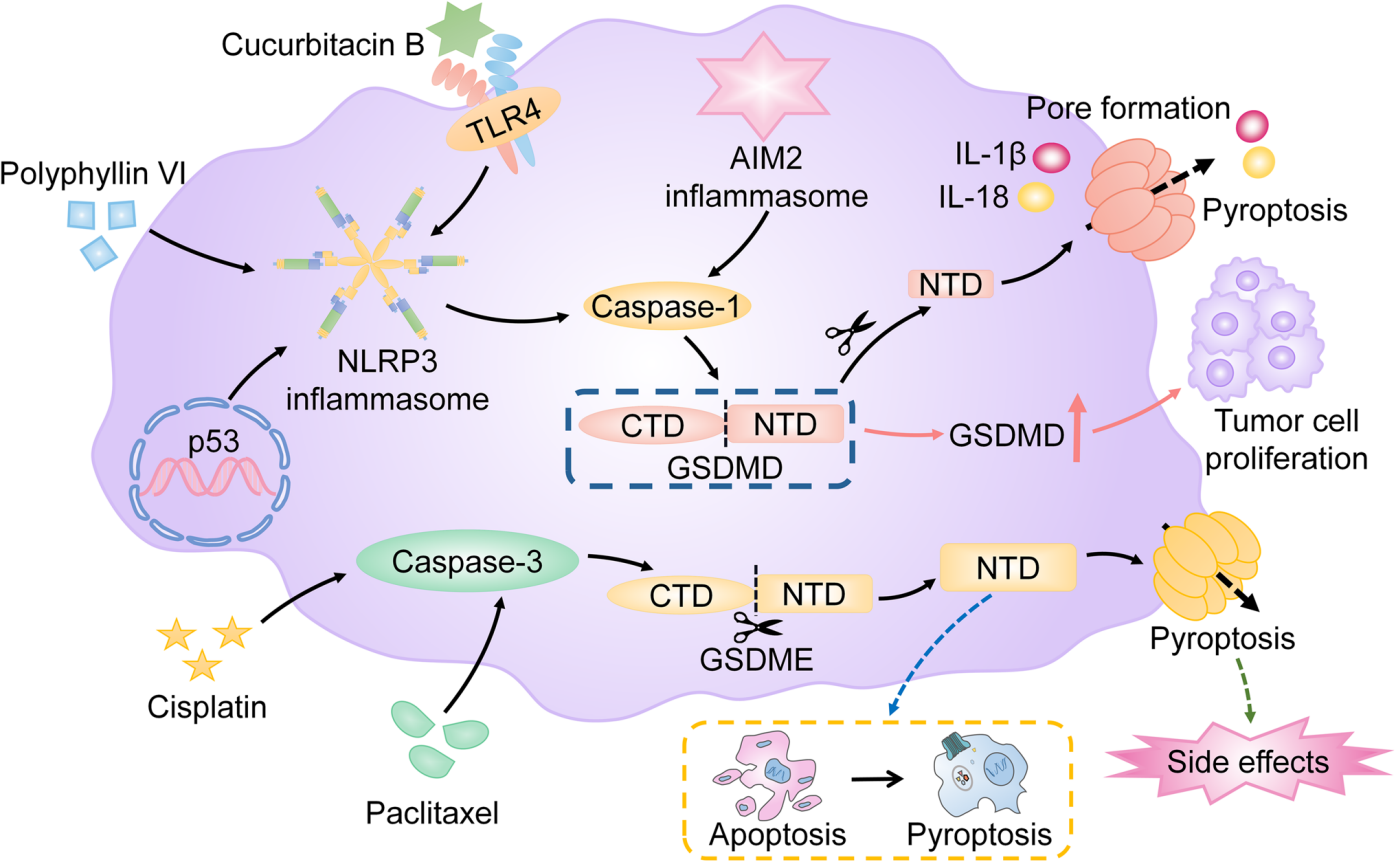
Pyroptosis in lung cancer. Increased GSDMD contributes to the proliferation of NSCLC cells. However, many potent anti-tumor drugs, such as polyphyllin VI and cucurbitacin B, induce NLRP3/caspase-1/GSDMD signaling pathway-mediated pyroptosis. Meanwhile, p53 induces pyroptosis to attenuate the proliferation of NSCLC cells by activating the NLRP3/ASC/caspase-1 signaling pathway. Additionally, chemotherapeutic drugs such as cisplatin and paclitaxel trigger caspase-3/GSDME-dependent pyroptosis to eliminate NSCLC cells and lead to the switch from apoptosis to pyroptosis in lung cancer cells. Notably, caspase-3/GSDME-dependent pyroptosis, which is mediated by cisplatin in normal tissues, contributes to the cisplatin-based side effects. Note: GSDMD, gasdermin D; NSCLC, non-small cell lung cancer; ASC, apoptosis-associated speck-like protein containing a caspase-activation and recruitment domain; GSDME, gasdermin E

Collectively, pyroptosis may have dual effects on lung cancer because it can promote both inflammation and cell death. However, the determinants of pyroptosis to induce inflammation or cell death remain unknown (Fig. 5).

### Pyroptosis and acute lung injury

Acute lung injury (ALI) is a life-threatening hypoxemic respiratory disease. Acute progressive pulmonary inflammation characterized by infiltration of inflammatory cells, increased permeability of the alveolar-capillary barrier, and acute diffuse alveolar edema are commonly found in ALI [10, 36].

Consistent excessive inflammation induced by alveolar macrophages (AMs) is the key to ALI [10]. Increasing studies indicate that pyroptosis of AMs may contribute to the development of ALI by mediating inflammation. It is reported that GSDMD-dependent pyroptosis is triggered by LPS in AMs via activating the TLR4/NLRP3/caspase-1 pathway, which leads to severe lung damage characterized by infiltration of inflammatory cells, pulmonary interstitial edema, hemorrhage, and alveolar wall thickness [27, 36]. Inhibition of NLRP3 inflammasome results in reduced pyroptosis and inflammatory cytokines such as IL-1β and IL-18 in AMs, which is followed by improved lung injury [27, 36, 82]. Additionally, the p38 MAPK signaling pathway, which mediates inflammation, is shown to participate in the pyroptosis of AMs in ALI by mediating the activation of NLRP3 inflammasome. It is reported that a selective inhibitor of the p38 MAPK signaling pathway, SB203580, suppresses the NLRP3/caspase-1-dependent pyroptosis in AMs, which results in the amelioration of lung inflammation [35]. Accordingly, NLRP3/caspase-1/GSDMD-mediated pyroptosis of AMs may participate in the development of ALI. Additionally, pyroptosis mediated by anthrax lethal toxin-activated NLRP1 inflammasome in AMs promotes lung inflammation and results in the exacerbation of ALI [32]. Furthermore, growing evidence suggests that pyroptosis may also trigger the enhanced inflammatory response in ALI by generating pyroptotic bodies (PyrBDs), which are mainly derived from pyroptotic AMs [52]. PyrBDs, which contain substantial DAMPs, trigger vascular interstitial edema and neutrophil recruitment [52].

**Fig. 6:**
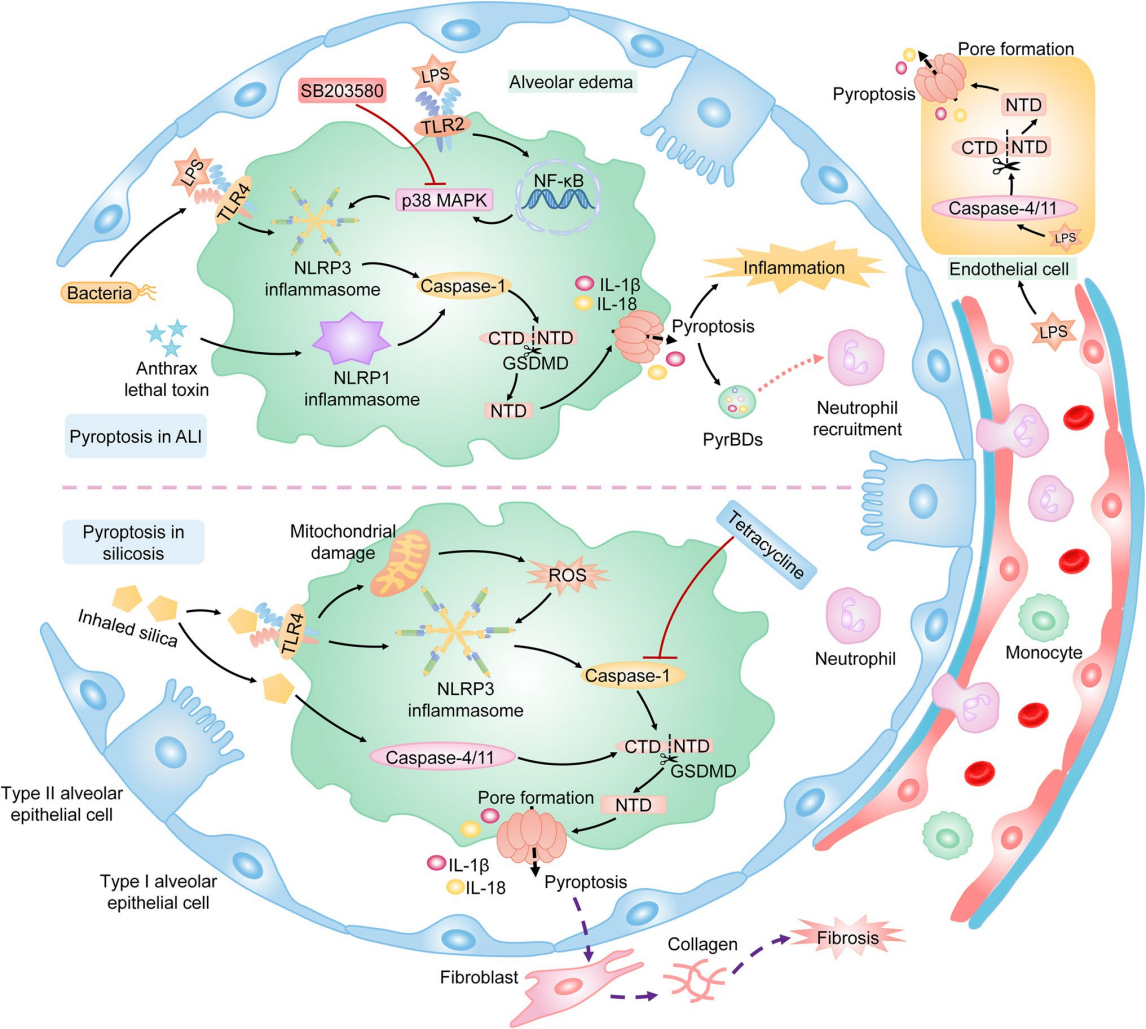
Pyroptosis in ALI and silicosis. Exposure to LPS, which is an important component of bacteria, is a common cause of ALI. LPS binds to TLR4 and activates the NLRP3 inflammasome. Meanwhile, LPS activates the p38 MAPK pathway via the TLR2/NF-κB signaling pathway, which results in the activation of the NLRP3 inflammasome. The activated NLRP3 inflammasome excites caspase-1, and active caspase-1 sequentially cleaves GSDMD. Finally, GSDMD-dependent pyroptosis of AMs occurs, which leads to consistent excessive alveolar inflammation and diffuse alveolar edema. Additionally, the lethal toxin-activated NLRP1 inflammasome induces pyroptosis of AMs, contributing to the development of ALI. Furthermore, pyroptosis of AMs aggravates ALI by releasing caspase-1-dependent PyrBDs, which trigger the vascular interstitial edema and neutrophil recruitment. In addition, LPS circulating in the blood triggers pyroptosis of pulmonary endothelial cells via activating the caspase-4/11/GSDMD pathway. Inhaled silica activates the NLRP3 inflammasome, which is dependent or independent of ROS. Activated NLRP3 inflammasome processes caspase-1 which cleaves GSDMD. Additionally, silica activates caspase-4/11, which leads to the release of GSDMD-NTD. Consequently, GSDMD-mediated pyroptosis occurs in AMs and results in chronic inflammation and pulmonary fibrosis. Note: ALI, acute lung injury; LPS, lipopolysaccharide; GSDMD, gasdermin D; AMs, alveolar macrophages; MAPK, mitogen-activated protein kinase; NF-κB, nuclear factor kappa-B; PyrBDs, pyroptotic bodies

In addition, loss of pulmonary endothelial integrity, which controls lung vascular permeability, is important in the pathogenesis of ALI [44]. The pulmonary endothelial cells are invariably exposed to LPS or other pathogens circulating in the blood. Therefore, pyroptosis of pulmonary endothelial cells may play an important role in ALI. It is shown that caspase-11/GSDMD-mediated pyroptosis in pulmonary endothelial cells leads to pulmonary edema, while the endothelial-specific deletion of caspase-11 reverses pulmonary inflammation and lung damage [9] (Fig. 6).

### Pyroptosis and silicosis

Silicosis is considered an occupational lung disease and is characterized by diffuse pulmonary fibrosis and silicon nodules [34]. AMs, which are the first cells that engulf inhaled silica particles, play a considerably essential role in the progression of silicosis [51, 66]. The phagocytosis of silica causes the release of cytokines such as IL-1β and IL-18 from AMs [51]. IL-1β and IL-18 are key molecules in regulating pulmonary inflammation and fibrosis, and the release of these two cytokines is correlated with pyroptosis [2]. Therefore, pyroptosis of AMs may play an important role in silicosis. Recently, it is shown that GSDMD-dependent pyroptosis is triggered in silica-treated AMs by activating the TLR4/NLRP3/caspase-1/GSDMD pathway, which results in aggravated pulmonary inflammation and diffuses silicon nodules [66, 83]. Blocking pyroptosis of macrophages by inhibiting the NLRP3 inflammasome or caspase-1 reduces silica-mediated pulmonary inflammation and fibrosis [50, 83]. Meanwhile, silica induces the generation of ROS, which is important in the activation of the NLRP3/caspase-1 signaling pathway to accelerate pyroptosis in AMs [83]. Additionally, it is shown that caspase-11 is upregulated in mice exposed to silica [66]. Inhibition of caspase-11 reduces GSDMD-induced pyroptosis in silica-treated macrophages [66]. Therefore, it is suggested that caspase-4/5/11/GSDMD-induced pyroptosis is involved in the development of silicosis. However, the specific mechanism is unclear (Fig. 6).

### Pyroptosis and pulmonary hypertension

Pulmonary hypertension (PH) is characterized by pulmonary vascular remodeling and increased right ventricular systolic pressure (RVSP) [22, 78, 88].

Accumulating evidence suggests that abnormal proliferation of pulmonary artery smooth muscle cells (PASMCs) induced by inflammation results in pulmonary vascular remodeling [22, 71, 88]. Glioma-associated oncogene family zinc finger 1 (GLI1) is a transcription factor that mediates cancer cell proliferation [81]. It is reported that GLI1 induces pyroptosis of PASMCs under hypoxic conditions by upregulating the expression of ASC, which results in vascular remodeling and increased RVSP [22]. Whereas, inhibition of GLI1 reduces pyroptosis of PASMCs and alleviates the progression of hypoxia-induced PH [22]. Additionally, PD-L1 is a transmembrane protein correlated to GSDMC-induced pyroptosis [24]. It is shown that PD-L1 triggers pyroptosis of PASMCs and sequentially contributes to hypoxia-induced PH [88]. However, the specific mechanism of the PD-L1-induced pyroptosis in PASMCs is unclear. Additionally, caspase-1 is an important molecule in the canonical inflammasome-induced pathway of pyroptosis. It is reported that caspase-1 induces the abnormal proliferation of PASMCs in hypoxia by upregulating the IL-1β/IL-18/IL-6/STAT3 pathway, which causes increased pulmonary arterial pressure [71]. Collectively, pyroptosis of PASMCs is highlighted in the progression of hypoxia-induced PH.

In addition, the pulmonary arterial endothelial cells (PAECs) dysfunction caused by enhanced vascular inflammation is an important cause of PH [69]. Wu et al. have elucidated that GSDMD-dependent pyroptosis is triggered by the activation of caspase-4/11 in TNF-α-treated PAECs. Meanwhile, caspase-4 enhances pyroptosis in PAECs by activating the caspase-3/GSDME pathway. Caspase-4/5-mediated pyroptosis in PAECs leads to pulmonary vascular resistance and increased RVSP [78]. While inhibition of caspase-4/11 ameliorates the development of PH and vascular remodeling [78].

**Fig. 7:**
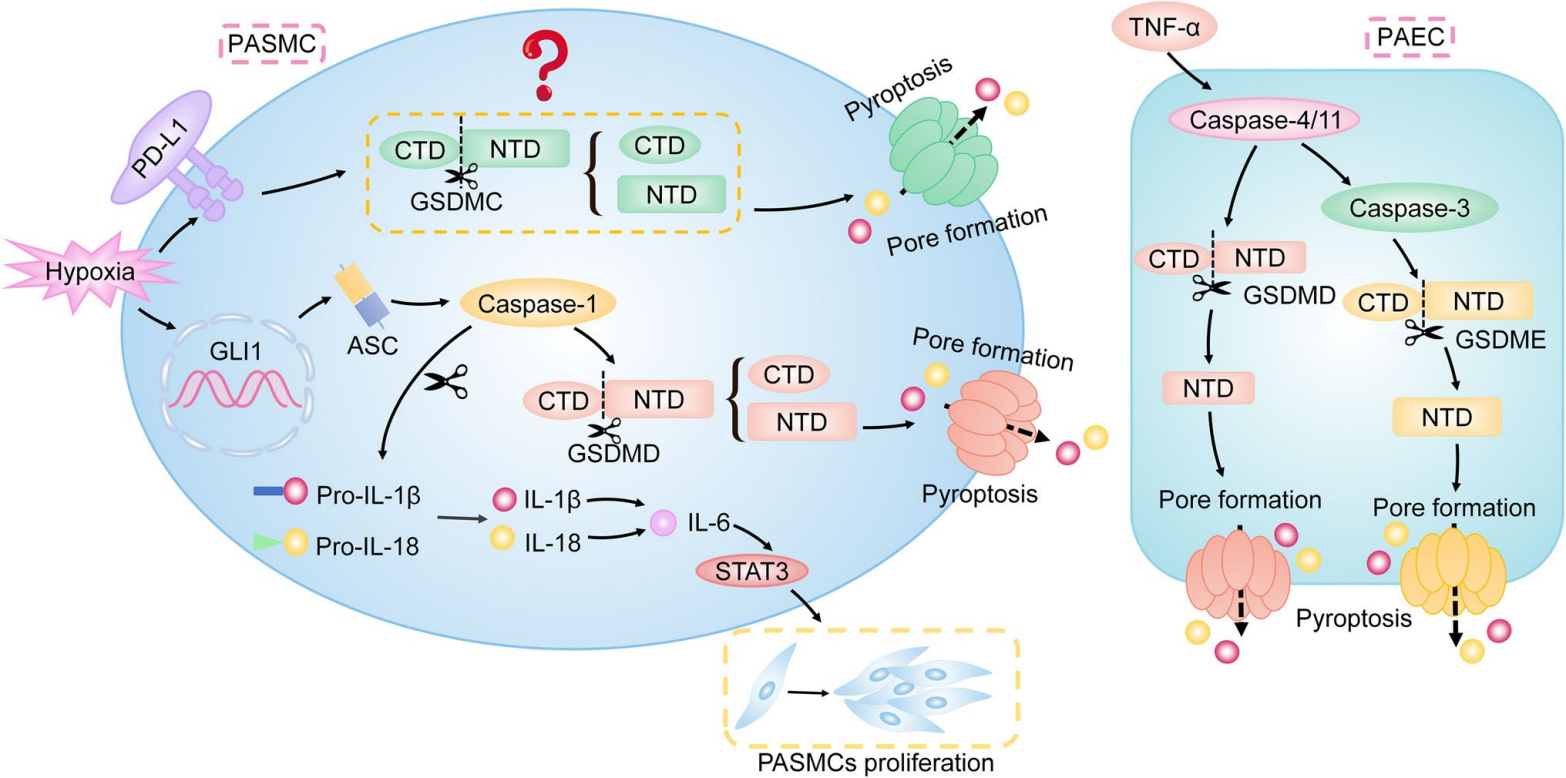
Pyroptosis in PH. The abnormal proliferation of PASMCs leads to pulmonary vascular remodeling. PD-L1 triggers pyroptosis of PASMCs, which contributes to hypoxia-induced PH. Though PD-L1 induces GSDMC-dependent pyroptosis, the specific mechanism of PD-L1-induced pyroptosis in PASMCs is unclear. Additionally, GLI1 induces pyroptosis of PASMCs under hypoxic conditions by increasing the level of ASC, which results in vascular remodeling. Meanwhile, increased IL-1β or IL-18 mediated by caspase-1 upregulates IL-6 to activate STAT3, which contributes to the abnormal proliferation of PASMCs. In addition, TNF-α is a classic pro-PH factor and causes PAECs dysfunction, which is an important cause of PH. It activates the caspase-4/11/GSDMD pathway to induce pyroptosis of PAECs. Meanwhile, caspase-4 also enhances pyroptosis in PAECs by activating the caspase-3/GSDME pathway. Consequently, pyroptosis of PAECs promotes pulmonary vascular resistance. Note: PH, pulmonary hypertension; PASMCs, pulmonary arterial smooth muscle cells; PD-L1, programmed death-ligand 1; GLI1, glioma-associated oncogene homolog 1; ASC, apoptosis-associated speck-like protein containing a caspase-activation and recruitment domain; STAT3, signal transducer and activator of transcription 3; TNF-α, tumor necrosis factor-alpha; PAECs, pulmonary arterial endothelial cells

Accordingly, pyroptosis is involved in the pathogenesis of PH (Fig. 7).

### Pyroptosis and cystic fibrosis

Cystic fibrosis (CF) is an autosomal recessive disorder attributed to the mutations of a gene that encodes the cystic fibrosis transmembrane conductance regulator (CFTR) protein [42]. Patients with CF suffer from recurrent and chronic respiratory infections and inflammation, which results in airway obstruction and even respiratory failure [6].

The deficiency of CFTR leads to reduced efflux chloride in epithelial cells, which contributes to bacterial proliferation and excessive inflammation in CF by forming a thick and viscous mucus layer [20]. Recently, it is reported that increased cytosolic chloride caused by dysfunction of CFTR induces the activation of the NLRP3 inflammasome in monocytes by upregulating the expression of P2X7R, which contributes to inflammation in CF. While the CFTR modulators restore the CFTR expression and reduce inflammation in patients with CF by blocking the P2X7R/NLRP3/caspase-1 axis [20]. Additionally, the absence of CFTR leads to the upregulation of epithelial sodium channel (ENaC), which may be partially responsible for the recurrent respiratory infections and chronic inflammation in CF [61]. Recently, it has been reported that the increased activity of ENaCs activates NLRP3/caspase-1 signaling pathway in human BECs with CF-associated mutations by directly increasing potassium efflux, which enhances excessive inflammation in CF [61]. The NLRP3/caspase-1 pathway is important in pyroptosis. Therefore, these results imply a potential role of NLRP3/caspase-1-dependent pyroptosis in CF. However, whether pyroptosis is involved in the pathogenesis of CF is still unknown.

Additionally, *Pseudomonas aeruginosa* is a common cause of airway inflammation and pulmonary tissue damage in CF. It is shown that *P. aeruginosa* can activate NLRC4 inflammasome, NLRP3 inflammasome, and caspase-11 [1]. Meanwhile, caspase-11-dependent pyroptosis is triggered in response to *P. aeruginosa* infection, suggesting that pyroptosis may participate in the inflammatory response against *P. aeruginosa* in CF patients. However, the specific mechanism is unclear.

### Pyroptosis and granulomatous diseases in the respiratory system

Inflammation is the main cause of granulomatous diseases [48]. Accumulating evidence suggests that pyroptosis may play a role in pulmonary granulomatous diseases such as tuberculosis (TB) and sarcoidosis [5, 53].

#### Tuberculosis

TB is a chronic inflammatory disease characterized by caseous necrotizing granuloma formation [40], which remains the leading cause of death worldwide [53]. The pathogen of TB is *Mycobacterium tuberculosis* (Mtb), which causes a host inflammatory response via complex mechanisms [54]. Studies show that pyroptosis of macrophages may play an important role in host resistance to Mtb infection by mediating inflammatory cytokines downstream. It is shown that EST12, which is a protein secreted by Mtb, interacts with receptors for activated C kinase 1 and sequentially induces the NLRP3/GSDMD-mediated pyroptosis to enhance mycobacterial clearance. While the EST12-deficient Mtb strain causes decreased pyroptosis and escapes the clearance of host cells in vitro and vivo [53]. Meanwhile, another study demonstrates that Mtb inhibits pyroptosis of macrophages by producing PknF, which is a phosphokinase of Mtb. Pkn inhibits the activation of the NLRP3 inflammasome by suppressing the potassium efflux and generation of ROS. The Pkn-induced inhibition of NLRP3/caspase-1/GSDMD-dependent pyroptosis then allows Mtb to evade the clearance of the host cell [55]. In addition to the NLRP3 inflammasome, the AIM2 inflammasome is highlighted in the host inflammatory response against Mtb. It is reported that Mtb inhibits the activation of AIM2 inflammasome to escape host clearance [62]. Meanwhile, AIM2-deficient mice are more susceptible to Mtb [58]. This research indicates that AIM2 inflammasome may take a protective effect against Mtb by regulating downstream inflammatory cytokines. AIM2 inflammasome is an important molecule in pyroptosis. Nonetheless, whether pyroptosis participates in the AIM2 inflammasome-mediated host inflammatory response against Mtb is unclear.

However, excessive pyroptosis induced by Mtb may lead to augmented destruction of the lung tissue as well as the dissemination of Mtb [4, 19, 40]. It is reported that the Mtb-induced plasma membrane rupture contributes to the NLRP3/caspase-1/GSDMD-dependent pyroptosis by inducing potassium efflux, which leads to bacterial growth and spread [4]. Additionally, a growing body of evidence suggests that the Mtb can trigger pyroptosis to promote the dissemination of bacteria via mediating ERS. Mtb-induced ERS leads to the upregulation of TXNIP by activating the PERK/eIF2α/CHOP signaling pathway [19]. TXNIP plays an important role in the activation of the NLRP3 inflammasome. It has been shown that inhibition of the PERK/eIF2α/TXNIP/NLRP3/caspase-1/GSDMD-mediated pyroptosis in Mtb-infected macrophages alleviates the lung tissue damage and restricts the spread of Mtb [19, 40].

**Fig. 8:**
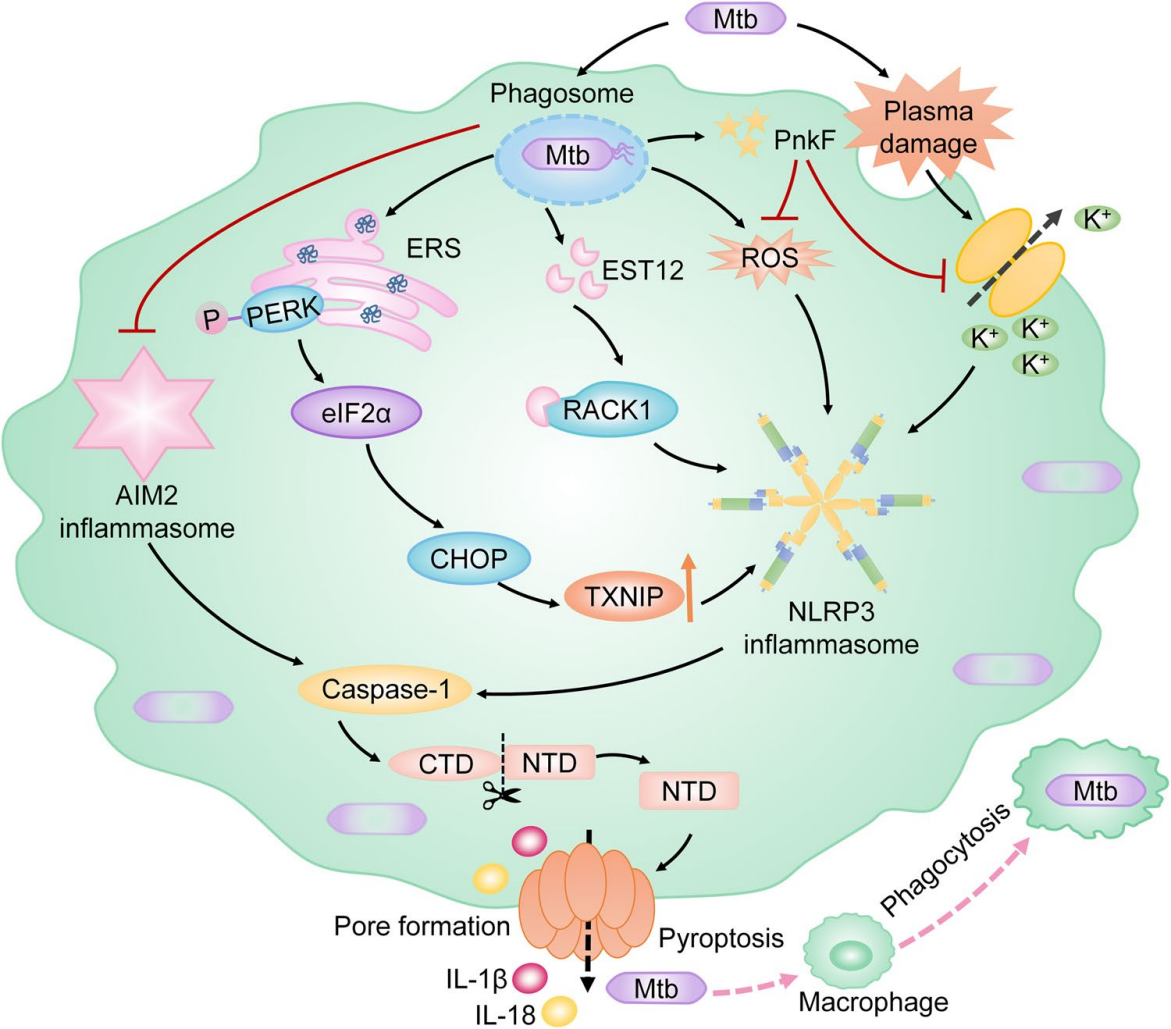
Pyroptosis in TB. Pyroptosis can promote the host clearance of Mtb. When Mtb invades the host, it is phagocytosed by macrophages to form a phagosome, which activates inflammatory response and restricts the growth of Mtb. EST12, which is secreted by Mtb, triggers pyroptosis through activating the RACK1/NLRP3/caspase-1/GSDMD signaling pathway, which improves the clearance of intracellular Mtb. Additionally, Mtb produces PnkF to suppress the potassium efflux and generation of ROS. Sequentially, the NLRP3/caspase-1/GSDMD-dependent pyroptosis is inhibited, which leads to the Mtb escaping from host clearance. Furthermore, the phagosome of Mtb inhibits the activation of the AIM2 inflammasome which is an important molecule in the pyroptotic pathway by an unknown mechanism to evade the host elimination. Additionally, Mtb-induced pyroptosis can trigger the dissemination of bacteria. Phagocytosed Mtb causes ERS which leads to the upregulated TXNIP via activating the PERK/eIF2α/CHOP pathway. Increased TXNIP leads to the activation of NLRP3 inflammasome. Meanwhile, Mtb triggers the activation of the NLRP3 inflammasome by inducing plasma damage. The NLRP3/caspase-1/GSDMD-dependent pyroptosis then occurs and results in the growth and spread of Mtb. Note: TB, tuberculosis; Mtb, *Mycobacterium tuberculosis*; AIM2, absent in melanoma 2; RACK1, receptor for activated C kinase 1; GSDMD, gasdermin D; ERS, endoplasmic reticulum stress; PERK, protein kinase R-like endoplasmic reticulum kinase; TXNIP, thioredoxin interacting protein; eIF2α, eukaryotic translation-initiation factor 2α; CHOP, CCAAT-enhancer-binding protein homologous protein

In summary, pyroptosis plays dual roles in TB. On the one hand, it participates in the host defense against Mtb infection. On the other hand, excessive pyroptosis induced by Mtb contributes to lung tissue damage and the spread of bacteria. However, the specific mechanism that determines the role of pyroptosis in TB remains unclear (Fig. 8).

#### Sarcoidosis

Sarcoidosis is an inflammatory disease characterized by the formation of non-necrotizing epithelioid granulomas [5]. IL-18 and IL-1β play a potential role in sarcoidosis [26]. The activation of the NLRP3 inflammasome contributes to the maturation and secretion of IL-18 and IL-1β. It is reported that downregulated mRNA levels of miR-223, a direct downregulator of the NLRP3 inflammasome, induce the increased expression of IL-1β in AMs from sarcoidosis patients than in healthy volunteers [26]. Meanwhile, in trehalose 6,6-dimycolate-treated mice, an NLRP3 inflammasome inhibitor reduces IL-1β, which is followed by a reduced number of pulmonary granuloma formation [26]. Additionally, serum amyloid A (SAA), which is highly expressed by macrophages in sarcoidosis granuloma, may play an important role in sarcoidosis via modulating the production of IL-18 and TNF [8]. It is suggested that SAA may activate the NLRP3 inflammasome as a DAMP to participate in the pathogenesis of sarcoidosis [26]. Collectively, the NLRP3 inflammasome may play a critical role in sarcoidosis. However, whether pyroptosis participates in sarcoidosis remains unknown.

## Conclusion

Pyroptosis is a novel regulated cell death induced by the GSDM family, which is usually accompanied by the release of active pro-inflammatory cytokines such as IL-1β and IL-18. There are several pathways involved in pyroptosis, such as the canonical inflammasome-induced pathway, non-canonical inflammasome-induced pathway, caspase-1/3/6/7/GSDMB pathway, caspase-8/GSDMC pathway, caspase-8/GSDMD pathway, and caspase-3/GSEME pathway. Although GSDMA is engaged in pyroptosis, its upstream effectors remain unclear.

Pyroptosis may be involved in inflammation-related respiratory diseases such as asthma, COPD, lung cancer, ALI, silicosis, PH, and TB, in which the NLRP3 inflammasome-induced pathway is mostly highlighted. Pyroptosis contributes to the deterioration of asthma, COPD, ALI, silicosis, and PH. In addition, pyroptosis appears to have dual effects in lung cancer and TB, while the molecules that determine the specific roles of pyroptosis in lung cancer and TB remain unclear. Furthermore, how the GSDME, as an executioner of pyroptosis, switches caspase-3-induced apoptosis to pyroptosis is not clear in lung cancer. Additionally, whether pyroptosis participates in CF and sarcoidosis or not is largely unknown, though the activation of NLRP3 inflammasome is found in CF and sarcoidosis. Collectively, the specific mechanisms of pyroptosis in inflammation-related respiratory diseases remain unclear. More studies on pyroptosis in inflammatory respiratory diseases are warranted in the future.

## Data Availability

Not applicable.

## Abbreviations

AHR: Airway hyperresponsiveness
AIM: Absent in melanoma
ALI: Acute lung injury
ALRs: Absent in melanoma-like receptors
AMs: Alveolar macrophages
ASC: Apoptosis-associated speck-like protein containing a caspase-activation and recruitment domain
ATP: Adenosine triphosphate
BECs: Bronchial epithelial cells
CF: Cystic fibrosis
CFTR: Cystic fibrosis transmembrane conductance regulator
CHOP: CCAAT-enhancer-binding protein homologous protein
COPD: Chronic obstructive pulmonary disease
CS: Cigarette smoke
CSE: Cigarette smoke extract
CTD: C-terminal domain
DAMPs: Danger-associated molecular patterns
dsDNA: Double-stranded DNA
eIF2α: Eukaryotic translation-initiation factor 2α
ENaC: Epithelial sodium channel
ERS: Endoplasmic reticulum stress
GLI1: Glioma-associated oncogene family zinc finger 1
GSDM: Gasdermin
GSDMD: Gasdermin D
IFN: Interferon
IL: Interleukin
LPS: Lipopolysaccharide
Mtb: *Mycobacterium tuberculosis*
NEK7: NIMA-related kinase 7
NF-κB: Nuclear factor kappa-B
NLRC: Nucleotide-binding oligomerization domain-like receptor, caspase activation and recruitment domain-containing protein
NLRP: NOD-like receptor protein
NLRs: Nucleotide-binding domain and leucine-rich repeat containing receptors
NSCLC: Non-small cell lung cancer
NTD: N-terminal domain
OMVs: Outer membrane vesicles
PAECs: Pulmonary arterial endothelial cells
P. aeruginosa: Pseudomonas aeruginosa
PAMPs: Pathogen-associated molecular patterns
PASMCs: Pulmonary arterial smooth muscle cells
PCD: Programmed cell death
PD-L1: Programmed death-ligand 1
PERK: Protein kinase RNA-like ER kinase
PGE2: Prostaglandin E2
PH: Pulmonary hypertension
PRRs: Pattern-recognition receptors
PYD: Pyrin domain
PyrBDs: Pyroptotic bodies
RAGE: Receptor for advanced glycation end-products
ROS: Reactive oxygen species
RVSP: Right ventricular systolic pressure
STAT: Signal transducer and activator of transcription
SAA: Serum amyloid A
TB: Tuberculosis
TDI: Toluene diisocyanate
Th: T helper cell
TLRs: Toll-like receptors
TNF-α: Tumor necrosis factor alpha
TXNIP: Thioredoxin interacting protein
